# Analyzing cell-type-specific dynamics of metabolism in kidney repair

**DOI:** 10.1038/s42255-022-00615-8

**Published:** 2022-08-25

**Authors:** Gangqi Wang, Bram Heijs, Sarantos Kostidis, Ahmed Mahfouz, Rosalie G. J. Rietjens, Roel Bijkerk, Angela Koudijs, Loïs A. K. van der Pluijm, Cathelijne W. van den Berg, Sébastien J. Dumas, Peter Carmeliet, Martin Giera, Bernard M. van den Berg, Ton J. Rabelink

**Affiliations:** 1 Department of Internal Medicine (Nephrology) & Einthoven Laboratory of Vascular and Regenerative Medicine, Leiden University Medical Center, Leiden, the Netherlands; 2 The Novo Nordisk Foundation Center for Stem Cell Medicine (reNEW), Leiden University Medical Center, Leiden, the Netherlands; 3 Center of Proteomics and Metabolomics, Leiden University Medical Center, Leiden, the Netherlands; 4 Department of Human Genetics, Leiden University Medical Center, Leiden, the Netherlands; 5 Leiden Computational Biology Center, Leiden University Medical Center, Leiden, the Netherlands; 6 Delft Bioinformatics Lab, Delft University of Technology, Delft, the Netherlands; 7 Laboratory of Angiogenesis and Vascular Metabolism, Department of Oncology, KU Leuven and Center for Cancer Biology, VIB, Leuven, Belgium; 8 Laboratory of Angiogenesis and Vascular Heterogeneity, Department of Biomedicine, Aarhus University, Aarhus, Denmark

**Keywords:** Metabolomics, Metabolomics, Metabolism

## Abstract

A common drawback of metabolic analyses of complex biological samples is the inability to consider cell-to-cell heterogeneity in the context of an organ or tissue. To overcome this limitation, we present an advanced high-spatial-resolution metabolomics approach using matrix-assisted laser desorption/ionization mass spectrometry imaging (MALDI-MSI) combined with isotope tracing. This method allows mapping of cell-type-specific dynamic changes in central carbon metabolism in the context of a complex heterogeneous tissue architecture, such as the kidney. Combined with multiplexed immunofluorescence staining, this method can detect metabolic changes and nutrient partitioning in targeted cell types, as demonstrated in a bilateral renal ischemia–reperfusion injury (bIRI) experimental model. Our approach enables us to identify region-specific metabolic perturbations associated with the lesion and throughout recovery, including unexpected metabolic anomalies in cells with an apparently normal phenotype in the recovery phase. These findings may be relevant to an understanding of the homeostatic capacity of the kidney microenvironment. In sum, this method allows us to achieve resolution at the single-cell level in situ and hence to interpret cell-type-specific metabolic dynamics in the context of structure and metabolism of neighboring cells.

## Abstract (web summary)

Understanding dynamic metabolic changes in complex biological samples often overlooks heterogeneity in cell composition. Wang et al. combine mass spectrometry imaging, isotope tracing, and multiplexed immunofluorescence microscopy to study metabolic dynamics in the kidney upon ischemia–reperfusion.

## Main

Spatial omics methods are becoming an increasingly important means to provide insights into in situ tissue cellular heterogeneity and how cells interact and behave within their microenvironments. The aim of analyzing the entirety of cellular metabolites at single-cell resolution is challenging owing to enormous structural diversity, rapid turnover, and low analyte abundances in a limited sample volume. Previously, MALDI-MSI-based metabolomics methods have been proposed as a way to measure spatial distribution of metabolites and lipids at single-cell resolution^1,2^. To date, MALDI-MSI-based single-cell metabolomics studies have, however, focused on analyzing single-timepoint metabolic ‘snapshots,’ which provide valuable information on in situ metabolic heterogeneity but crucially lack insight into the dynamic component of cellular metabolism^3–5^; hence, there is a need for approaches that provide a truly comprehensive understanding of the interplay between biochemical alterations and cell-type-specific functions, metabolic fluxes, and dynamic interpretations. To this end, isotope tracing has been used to model dynamic metabolic processes using bulk metabolomics^6^, in which complete tissues are homogenized and reduced to a single metabolic profile, disregarding tissue and metabolic heterogeneity. The combination of isotope tracing and subsequent analysis by MALDI-MSI could be considered as an approach to assess metabolic dynamics in situ^7–9^. Here, we describe a platform based on high-spatial-resolution MALDI-MSI (5 × 5 µm^2^ pixel size) that uses isotope tracing to allow for in situ cell-type-specific dynamic metabolism measurements to uncover cell metabolism within the architecture of the tissue that the cells reside in. The average diameter of most cells in kidney tissue is around 10 µm, and the proposed method provides a single measurement at subcellular-level resolution because each cell has a size of approximately 4 pixels in MALDI-MSI images. To gain the most information from the measured area, all pixels, not just those segmented into individual cells, will be used for the cell-type-specific metabolomics analysis that we describe below.

**Fig. 1:**
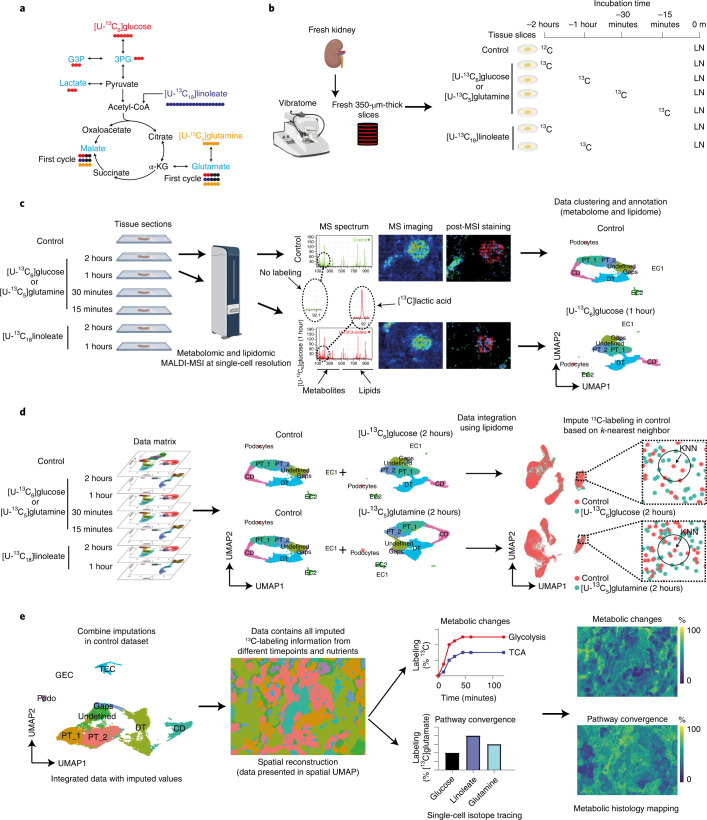
Workflow of cell-type-specific dynamic metabolic measurements and analysis. **a**, Overview of the traced isotopes in the primary carbon metabolism. The contributions of U-^13^C-labeled nutrients ([U-^13^C_6_]glucose, [U-^13^C_18_]linoleate, and [U-^13^C_5_]glutamine) to glycolytic and TCA intermediates (light blue) were traced. **b**, Fresh mouse kidney tissue was cut into 350-µm-thick slices using a vibratome. For ^13^C isotope tracing in tissue culture, different ^13^C-labeled nutrients were added to a well-defined medium described in the Methods section at different timepoints. Samples were quenched using liquid nitrogen (LN). **c**, The metabolome and lipidome were measured in all samples using MALDI-MSI at high spatial resolution (5 × 5 µm^2^ pixel size). MALDI-MSI data were preprocessed and transferred into a data matrix. Cell types were identified on the basis of lipid profiles and IF staining after MALDI-MSI. Images in **b** and **c** were created using Biorender. **d**, Cell-type-specific (phospho)lipid data were used to characterize various cell types. The lipid data were used for anchor-based data integration of the ‘control’ data matrix with data matrices of sections from ^13^C-isotope tracing measurements. We used KNN analysis to impute the molecular information contained in the ^13^C-labeling timecourse data matrices into the control data matrix. **e**, Establishment of the imputed dataset, in which each pixel contains all added ^13^C-labeling information from each timepoint and labeled nutrient. Dynamic metabolic calculations were performed on single pixels, including metabolic rates and pathway convergence. To visualize the heterogeneity in tissue metabolic dynamics, a series of pseudoimages, which were generated from calculated values, were created by tracing pixel coordinates back to the original spatial information from the MALDI-MSI analysis. α-KG, alpha-ketoglutaric acid; DT, distal tubules; CD, collecting ducts; PT, proximal tubules; EC, endothelial cells; GEC, glomerular endothelial cells; TEC, peritubular endothelial cells.

**Extended Data Fig. 1:**
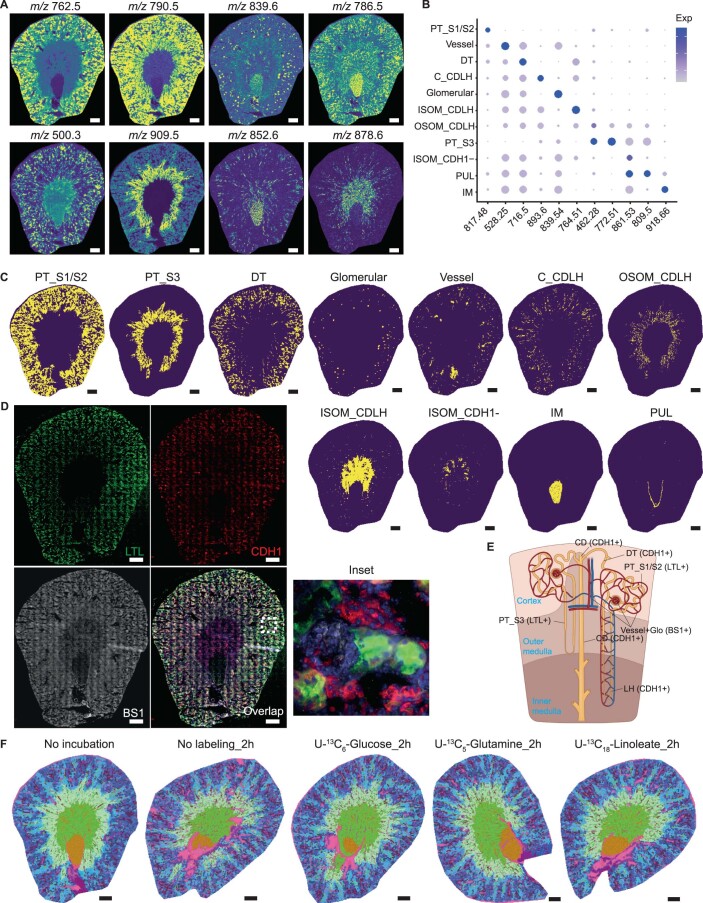
Lipid heterogeneity and stability in mouse kidneys. **a**, Lipid species distribution in a mouse kidney tissue sample as recorded by MALDI-MSI at 20 × 20 μm^2^ pixel-size. **b**, Dot plot displaying lipid expression of cluster-enriched signatures. **c**, Distribution of different renal cell clusters on tissue as identified in Fig. 2a. **d**, Immunofluorescent staining (*Lotus Tetragonolobus* Lectin (LTL, green), E-cadherin (CDH1, red) and BS1-lectin (gray)) after MALDI-MSI measurement. **e**, Nephron anatomy showing the identification of renal cell types based on immunofluorescent staining. **f**, Molecular histology of kidneys generated from integrated three-dimensional UMAP analysis of different datasets based on lipid profiles showing the specificity and stability of lipids profiles after introducing ^13^C-labeled nutrients for 2 hours. All scale bars = 500 µm. Abbreviations: *PT_S1/S2, cortical proximal tubular segments 1/2; PT_S3, outer stripe of outer medulla proximal tubular segment; DT, distal tubule; CDLH, collecting duct and loop of Henle; IM, inner medulla; ISOM, inner stripe of outer medulla; OSOM, outer stripe of outer medulla; PUL, pelvic urothelial lining*.

Our method uses ^13^C-labeled nutrients that allow tracing of the spatiotemporal incorporation of the ^13^C isotopes into the main intermediates of glycolysis and the tricarboxylic acid (TCA) cycle (Fig. 1a). Using different labeled nutrients allows not only visualization of dynamic changes over time, but also resolution of their contributions to specific metabolic pathways. To accomplish this, we used a tissue culture system in which 350-µm-thick vibratome slices of fresh mouse kidney^10^ (Fig. 1b) were incubated for up to 2 hours. During incubation, ^13^C-isotope-labeled nutrients were introduced in parallel to the tissue culture medium at selected timepoints, which led to efficient and biochemically meaningful labeling of metabolically active cells^11^. We could then measure metabolic changes in a non-steady state. Subsequently, tissue slices were quenched with liquid nitrogen and sectioned into 10-µm-thick sections, thaw-mounted on conductive glass slides, and coated with a chemical matrix for MALDI-MSI measurements. Negative-ion-mode MALDI-MSI at subcellular resolution (that is, pixel size of 5 × 5 µm^2^) was used to detect metabolites and (phospho)lipids^12^ (Extended Data Table 1) from the collected tissues. Following MALDI-MSI measurements, the sections were stained and subsequently imaged using multiplexed immunofluorescence (IF) microscopy for cell-type identification (Fig. 1c). By combining IF and spatial segmentation of the MALDI-MSI data (on the basis of uniform manifold approximation and projection (UMAP) of the lipid signals, in particular), cell-type-specific signatures were established using the following assumptions: (1) MALDI-MSI lipid profiles are cell-type specific and (2) major phospholipid species, important for cell typing, are mainly cell-membrane components^13^ and thus are stable during the 2 hours of isotopic labeling (Extended Data Fig. 1). Providing functional evidence for this approach, MALDI-MSI and molecular histology of tissues incubated with [U-^13^C]linoleate, [U-^13^C]glutamine, or [U-^13^C]glucose revealed similar distributions of lipid signatures for cell typing (Extended Data Fig. 1f). MALDI-MSI measurements were performed on sections at different timepoints and with different ^13^C-enriched nutrients to obtain the labeling datasets for each condition. Thus, in order to show all the dynamic metabolic changes within 1 pixel without batch effects, a two-step process was followed, as introduced by Stuart et al. in the Seurat package^14^: (1) single-pixel lipid profiles were used to identify anchors between two datasets; (2) ^13^C-labeled-metabolite production was imputed into the control dataset by transferring the abundance of all measured ^13^C-enriched metabolites from the labeling datasets using *k*-nearest neighbors (KNN) analysis (Fig. 1d). An imputed dataset containing the complete ^13^C-labeling information, representing the predicted values of mass isotopomers from the pixels with a similar lipid profile for each tracing experiment, from different timepoints and nutrients could thus be established. Dynamic metabolic calculations, including metabolic changes and pathway convergence, and assessment of ^13^C enrichment of isotopologues were performed on single-pixel data. Finally, the in situ heterogeneity of metabolic dynamics was visualized in pseudoimages generated from the calculated values (Fig. 1e).

**Extended Data Fig. 2:**
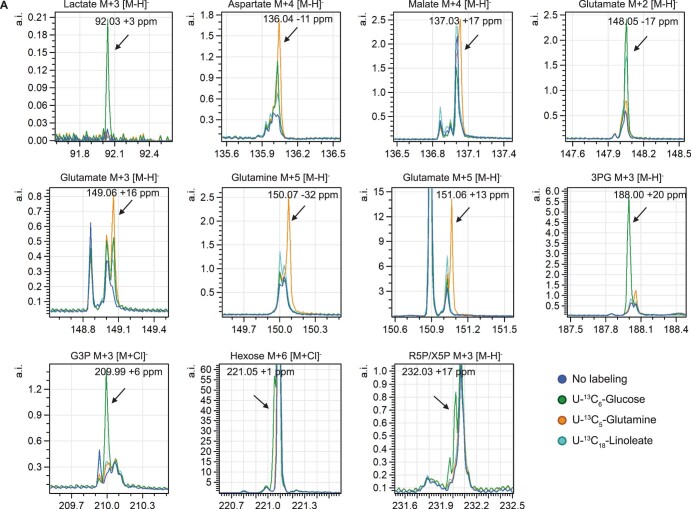
Mass spectrum of measured ^13^C-labeled Metabolites. Mass spectra obtained from ^13^C-labeled- and control samples measured by MALDI-MSI at 5 × 5 µm^2^ pixel size. Graphs show presence of various ^13^C-labeled metabolites.

**Extended Data Fig. 3:**
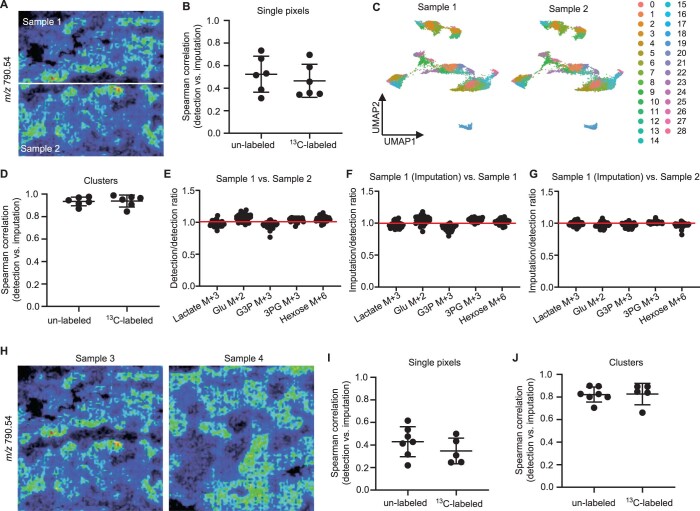
Validation of the imputation performance – ‘leave-one factor-out’ cross-validation. **a**, MALDI-MSI data from a single kidney measurement, which was incubated with U-^13^C_6_-glucose for 2 h, was bisected (into sample 1 and sample 2) and used to test the accuracy of the imputation. *m/*z features corresponding to metabolites were taken out from sample 1. Based on these lipid profiles, metabolite abundance of sample 2 was used to impute their abundance in sample 1. **b**, Spearman’s correlation analysis on the imputed and detected metabolites in kidney sample 1 based on all the pixels. Dots represent different metabolites. **c**, UMAP analysis on integrated MALDI-MSI data of the 2 samples and resulting 29 clusters. **d**, Spearman’s correlation analysis on the average value of each cluster between the imputed and detected metabolites abundance in kidney sample 1. **e**, The ratio of the ^13^C enrichment calculated from detected metabolites abundance in the 29 clusters between sample 1 and sample 2. Dots represent different clusters. **f**, The ratio of the ^13^C enrichment calculated from the imputed and detected metabolites abundance in the 29 cell clusters of kidney sample 1. Dots represent different clusters. **g**, The ratio of the ^13^C enrichment calculated from the imputed metabolites abundance of sample 1 from sample 2 and detected metabolites abundance of kidney sample 2 in the 29 cell clusters. Dots represent different clusters. **h**, MALDI-MSI data from measurements of two different kidneys, which were incubated with U-^13^C_6_-glucose for 2 h, were used to test the performance of the imputation between different measurements. **i**, Spearman’s correlation analysis on the imputed and detected metabolites production based on all the pixels. Dots represent different metabolites. **j**, Spearman’s correlation analysis on the average value of each cluster between the imputed and detected metabolites abundance. Dots represent different metabolites. All the performance test were done on 3 different imputations from different biological independent samples. Source data

**Extended Data Fig. 4:**
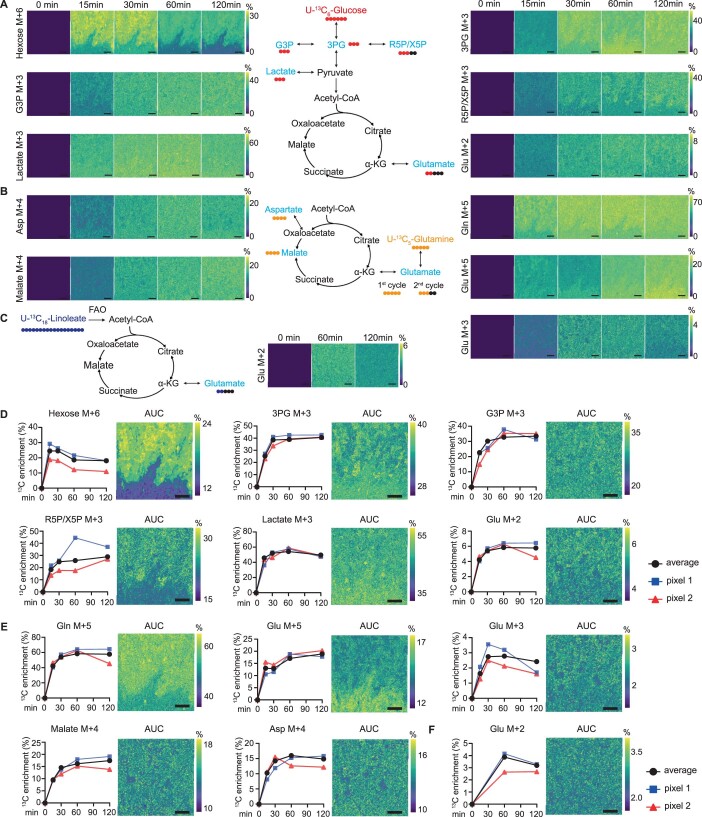
Metabolic dynamics measurements on mouse kidney. **a**, Pseudo-images showing the ^13^C enrichment of isotopologues of different metabolites over a time course (up to 2 h) of incubation with U-^13^C_6_-glucose. Color scale representing the ^13^C enrichment (%). Molecular histology and immunofluorescent staining on post-MALDI-MSI tissue are shown in Fig. 3a. **b**, Pseudo-images showing the ^13^C enrichment of isotopologues of different metabolites during incubation with U-^13^C_5_-glutamine for 2 h. Color scale representing the ^13^C enrichment (%). **c**, Pseudo-images showing the ^13^C enrichment of Glu M + 2 isotopologues during incubation with U-^13^C_18_-linoleate for 2 h. Color scale representing the ^13^C enrichment (%). **d**-**f**, Graphs showing the curve of ^13^C enrichment of isotopologues of metabolites over a time course (up to 2 h) with incubation of U-^13^C_6_-glucose (d), U-^13^C_5_-glutamine (e) and U-^13^C_18_-linoleate (f). Average values of all pixels and values from two representative pixels are shown. Images show the average ^13^C enrichment (area under curve (AUC) normalized to total time) of isotopologues measured at different timepoints. All scale bars = 200 µm. Abbreviations: *3PG, 3-phosphoglycerate; G3P, glycerol 3-phosphate; α-KG, alpha-ketoglutaric acid*. Source data

This approach allowed us to detect both endogenous and ^13^C-labeled metabolites from glycolysis and the TCA cycle, as well as branching metabolites, including hexose, glycerol-3-phosphate (G3P), 3-phosphoglycerate (3PG), ribose-5-phosphate/xylulose-5-phosphate (R5P/X5P), lactate, glutamate, glutamine, malate, aspartate, and linoleate (Extended Data Fig. 2). To validate metabolite imputation performance, a ‘leave-one-factor-out’ cross-validation^15^ was applied to a bisected MALDI-MSI dataset from a single kidney section after 2 hours of incubation with [U-^13^C_6_]glucose (Extended Data Fig. 3). The ‘new’ datasets (samples 1 and 2) created from the single MALDI-MSI measurement underwent identical sample handling and thus had comparable technical variation and metabolic activity (Extended Data Fig. 3a). The correlation between the imputed and measured metabolites at the single-pixel level was moderate (mean *r* = 0.49, *P* < 0.001, Extended Data Fig. 3b). To test imputation performance at the cell-type level, we divided all pixels into 29 clusters on the basis of their lipid profiles (Extended Data Fig. 3c). Following clustering, cell-type validation was performed using Spearman’s correlation analysis on the average value of each cluster. A very strong correlation (mean *r* = 0.94, *P* < 0.05) was observed between the imputed and detected values (Extended Data Fig. 3d). In addition, when comparing imputed and detected metabolite abundance, the average ^13^C enrichment of isotopologues in relation to endogenous metabolites in each cluster was highly accurate (ratio = 0.99 ± 0.04) (Extended Data Fig. 3e–g). We continued to test the MALDI-MSI data from two kidneys (samples 3 and 4) and found a strong correlation between imputed and detected metabolite intensity at the cluster level (mean *r* = 0.82, *P* < 0.001, Extended Data Fig. 3h–j). Following validation of the dynamic metabolism analysis, which revealed that the imputed cell-type-specific ^13^C-enrichment calculations were very reliable, pseudoimages were generated to show the spatial changes in dynamic metabolism within one tissue (Extended Data Fig. 4).

**Fig. 2:**
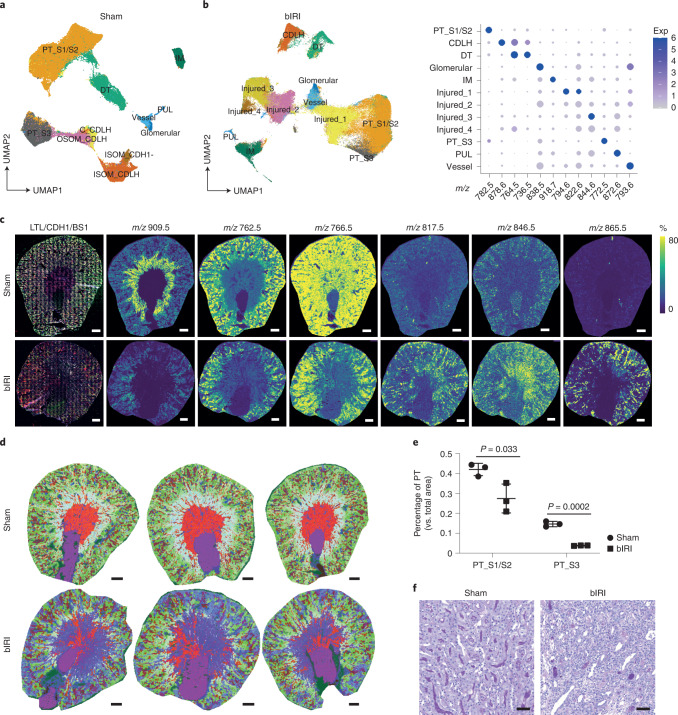
Lipid heterogeneity in mouse IRI kidneys. **a**, Lipid heterogeneity in mouse sham kidneys (*n* = 3) from which mice were opened up and closed similarly as bIRI mice but no ligations were performed, visualized in a two-dimensional (2D) UMAP plot of MALDI-MSI data (20 × 20 µm^2^ pixel size). **b**, Lipid heterogeneity in mouse bIRI kidneys (*n* = 3), visualized in a 2D UMAP plot of MALDI-MSI data at 20 × 20 µm^2^ pixel size. The dot plot displays lipid expression of cluster-enriched signatures. Exp, expression. **c**, IF staining (LTL, green), E-cadherin (CDH1, red), and BS1-lectin (gray)) of tissue that had been analyzed with MALDI-MSI. Representative images showing lipid species distributions in sham (*n* = 3) and bIRI (*n* = 3) kidneys, as recorded by MALDI-MSI (20 × 20 μm^2^ pixel size). Scale bars, 500 µm. **d**, Molecular histology of sham (*n* = 3) and bIRI (*n* = 3) kidneys generated from integrated three-dimensional (3D) UMAP analysis on the basis of lipid profiles. The color code represents the position of pixels in the 3D UMAP (UMAP1: red, UMAP2: green, UMAP3: blue). Scale bar, 500 µm. **e**, Comparison of PT areas between sham (*n* = 3) and bIRI (*n* = 3) kidneys. Two-tailed *t*-test was performed. **f**, PAS staining showing the tubular structures in the outer stripe outer medulla area of sham (*n* = 3) and bIRI (*n* = 3) kidneys. Scale bars, 50 µm. PT-S1/S2, cortical proximal tubular segments 1 and 2; PT-S3, outer stripe of outer medulla proximal tubular segment; DT, distal tubule; CDLH, collecting duct and loop of Henle; IM, inner medulla; OSOM, outer stripe of outer medulla; ISOM, inner stripe of outer medulla; PUL, pelvic urothelial lining. Source data

**Extended Data Fig. 5:**
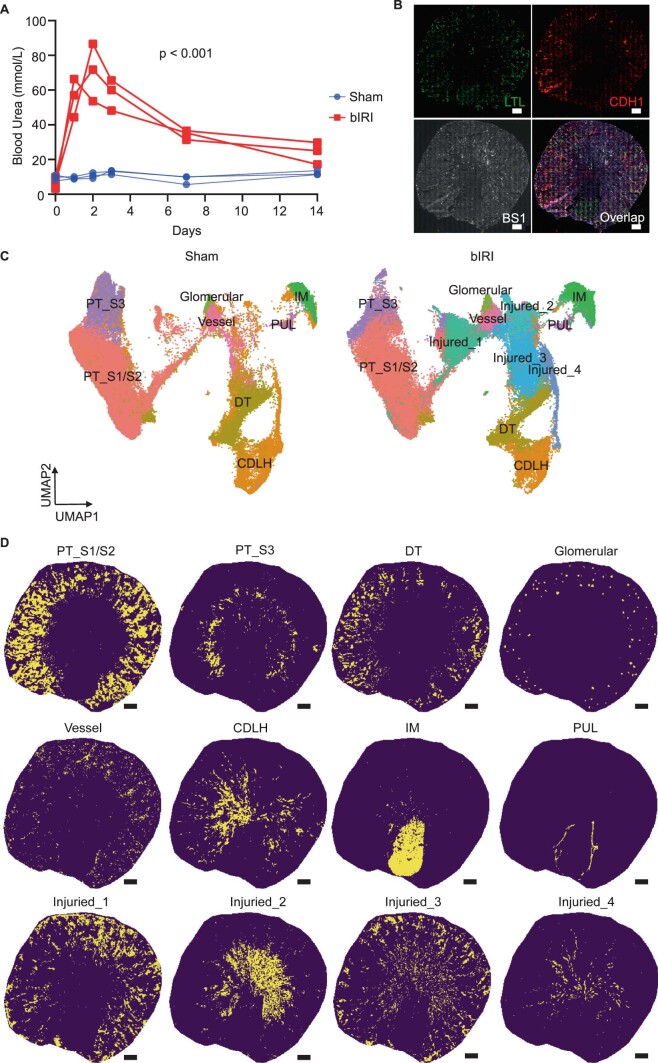
Lipid heterogeneity in mouse IRI kidneys. **a**, Comparison of blood urea levels at different timepoints after IRI surgery (n = 3 per group). Two tailed T test was performed on AUC. **b**, Immunofluorescent staining (*Lotus Tetragonolobus* Lectin (LTL, green), E-cadherin (CDH1, red) and BS1-lectin (gray)) after MALDI-MSI measurement of mouse IRI kidney. **c**, UMAP analysis on integrated lipidomics data of sham (n = 3) and bIRI (n = 3) mouse kidney samples. **d**, Distribution of different renal cell clusters on tissue as identified in Fig. 2b. All scale bars = 500 µm. Source data

To demonstrate how the method can be used, we induced moderate ischemic injury to mouse kidneys, similar to that observed in a kidney-transplantation setting. We then studied the tissue metabolism 2 weeks after the injury. At this timepoint, part of the initial proximal tubular epithelial injury has regenerated (Extended Data Fig. 5a) and has taken up a normal phenotype again (characterized by *Lotus tetragonolobus* lectin (LTL) positivity and kidney injury molecule-1 (KIM1) negativity), while other epithelial cells may still have a maladaptive inflammatory phenotype, as exemplified by vascular cell adhesion molecular 1 (VCAM1) expression in the presence of LTL, or may have lost the tubular epithelial phenotype all together (LTL negative)^16^. To visualize overall metabolic heterogeneity in whole kidney tissue sections, we used MALDI-MSI at a 20 × 20 µm^2^ pixel resolution, which spatially and molecularly resolved all major renal structures on the basis of their specific lipid signatures (Fig. 2a and Extended Data Fig. 1). Two weeks after kidney bIRI, we found additional clusters (Injured_1, Injured_2, Injured_3, and Injured_4) with unique lipid signatures that corresponded with loss of LTL and E-cadherin staining (Fig. 2a–c and Extended Data Fig. 5b–d). A loss of the healthy proximal tubule (PT) lipid signature and an increased presence of oxidized lipid species (*m/z* 865.5; Extended Data Table 1) were observed in the areas with persisting epithelial injury (Fig. 2c). We then generated spatial segmentation images using an integrated 3D UMAP analysis, which displayed the kidney molecular histology. These images showed the presence of ongoing tubular injury in both the cortex and outer medulla of bIRI kidneys (Fig. 2d). There was substantial loss of PT structures from the outer stripe of outer medulla (PT-S3) compared with the renal cortex (PT-S1/PT-S2) (Fig. 2e). These observations were also confirmed using a histopathological staining (Fig. 2f).

**Fig. 3:**
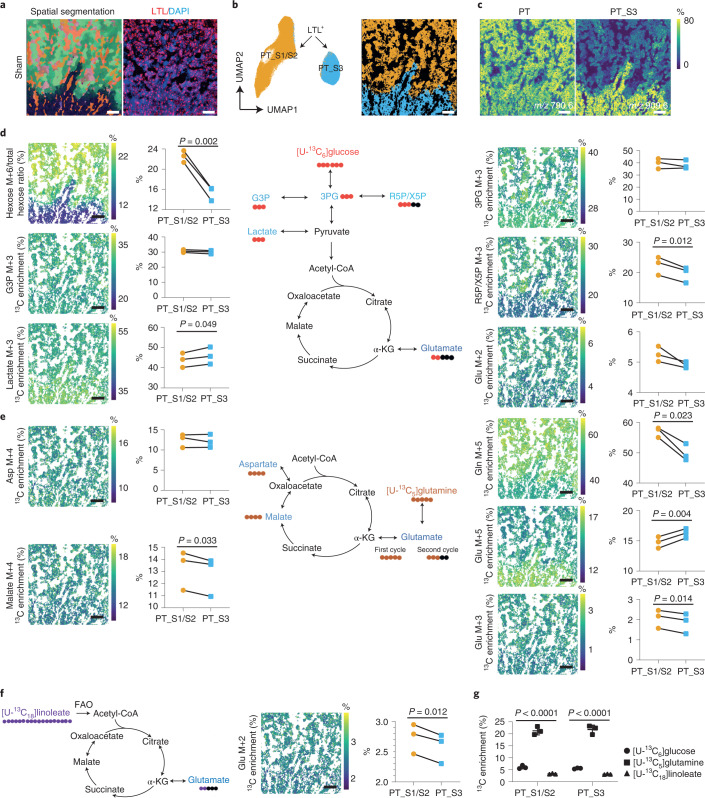
Dynamic metabolic measurements on sham kidney PT cells. **a**, Left, representative molecular histology of cortical and outer stripe of outer medulla areas of sham kidney (*n* = 3), generated from three-dimensional UMAP analysis on the basis of lipid profiles recorded by MALDI-MSI (5 × 5 μm^2^ pixel size). Right, LTL immunofluorescent staining on post-MALDI-MSI tissue (red). **b**, 2D UMAP plot and representative spatial segmentation showing lipid heterogeneity between LTL-positive proximal tubular cells from the cortical and outer stripe of outer medulla areas of sham kidneys (*n* = 3). **c**, Representative images showing lipid species distribution in the cortical and outer stripe of outer medulla areas of sham kidney (*n* = 3), as recorded by MALDI-MSI (5 × 5 μm^2^ pixel size). **d**, Dynamic metabolic measurements using [U-^13^C_6_]glucose on PT cells of sham kidneys. **e**, Dynamic metabolic measurements using [U-^13^C_5_]glutamine on PT cells of sham kidneys. **f**, Dynamic metabolic measurements using [U-^13^C_18_]linoleate on PT cells of sham kidneys. Images showing the average ^13^C enrichment of isotopologues on tissue. Graphs showing the comparison of the average ^13^C enrichment of isotopologues between PT S1/S2 and PT S3. The average ^13^C enrichment (area under curve (AUC) normalized to total time) of isotopologues were derived from [U-^13^C_6_]glucose or [U-^13^C_5_]glutamine measured at different timepoints (0, 15, 30, 60, and 120 minutes) or [U-^13^C_18_]linoleate measured at different timepoints (0, 60, and 120 minutes). Charts showing the traced isotopes and their derived isotopologues. Two-tailed paired *t*-test was performed. All scale bars, 200 µm. **g**, Direct carbon contribution of different nutrients to glutamate at the 2-hour timepoint, as measured from the glutamate isotopologues M+2, M+3, and M+5. One-way analysis of variance (ANOVA) was performed (*n* = 3). Glc, glucose; 3PG, 3-phosphoglycerate; G3P, glycerol-3-phosphate; R5P/X5P, ribulose-5-phosphate/xylulose-5-phosphate; α-KG, alpha-ketoglutaric acid; Glu, glutamate; Gln, Glutamine; Asp, aspartate. Source data

Considering that a tissue’s microenvironment is an important cue for cell-specific metabolism within that tissue, we next used high-spatial-resolution MALDI-MSI measurements with a pixel size at the subcellular level (5 × 5 µm^2^) to investigate the metabolic heterogeneity in the PT of the kidney cortex and outer stripe of outer medulla and their response to injury. Following MALDI-MSI, IF staining was conducted to identify cell types (Fig. 3a). We could distinguish two PT-S1 and PT-S2 cortex segments and one medullary PT-S3 segment on the basis of their lipid profiles (Fig. 3a–c). PT-S1 and PT-S2 were combined for subsequent analyses because we could not annotate them on the basis of LTL staining or their location. Analysis of the isotopologues showed that PT-S3 had lower hexose M+6 ([U-^13^C_6_]hexose) and higher lactate M+3 ([^13^C_3_]lactate) enrichment than did PT-S1/PT-S2 (Fig. 3d). In addition, we found lower enrichment of R5P/X5P M+3 (glycolysis branch) as well as lower enrichment of glutamate ([^13^C_2_]glutamate; Glu M+2) from glucose carbons entering the TCA cycle in PT-S3 (Fig. 3d). To further evaluate the use of ^13^C-labeled nutrients in the TCA cycle, we measured the percentage enrichment of [U-^13^C_5_]glutamine-derived isotopologues, [U-^13^C_5_]glutamate (Glu M+5), [^13^C_4_]malate (malate M+4), and [^13^C_3_]glutamate (Glu M+3), all of which are intermediates of one oxidative TCA cycle. There was lower [U-^13^C_5_]glutamine and higher [U-^13^C_5_]glutamate enrichment in PT-S3 cells than in PT-S1/PT-S2 cells (Fig. 3e). Notably, the enrichment of [^13^C_4_]malate and [^13^C_3_]glutamate were relatively lower in PT-S3 than in PT-S1/PT-S2 from the same sample, pointing to a lower contribution of both [U-^13^C_5_]glutamine and [^13^C_5_]glutamate to the oxidative TCA cycle in PT-S3 (Fig. 3e). The contribution of [U-^13^C_18_]linoleate to the [^13^C_2_]glutamate isotopologue through fatty acid oxidation was lower in PT-S3 as well (Fig. 3f). By combining these data, we found that [U-^13^C_5_]glutamine, rather than [U-^13^C_6_]glucose or [U-^13^C_18]_linoleate, is glutamate’s main carbon source in all PT areas (Fig. 3g). However, usage of glutamate for the oxidative TCA cycle differed between PT segments, as shown by glutamine carbon tracing to characteristic mass isotopomers (Fig. 3e). Together, these data show that, in the normal kidney, there is a marked heterogeneity among PT segments with respect to TCA metabolite consumption and glycolysis: the PT-S3 segment appears to be adapted to the low-oxygen-tension environment of the outer medulla. These observations correspond to previous studies using isolated or micro-dissected PTs^17,18^.

**Fig. 4:**
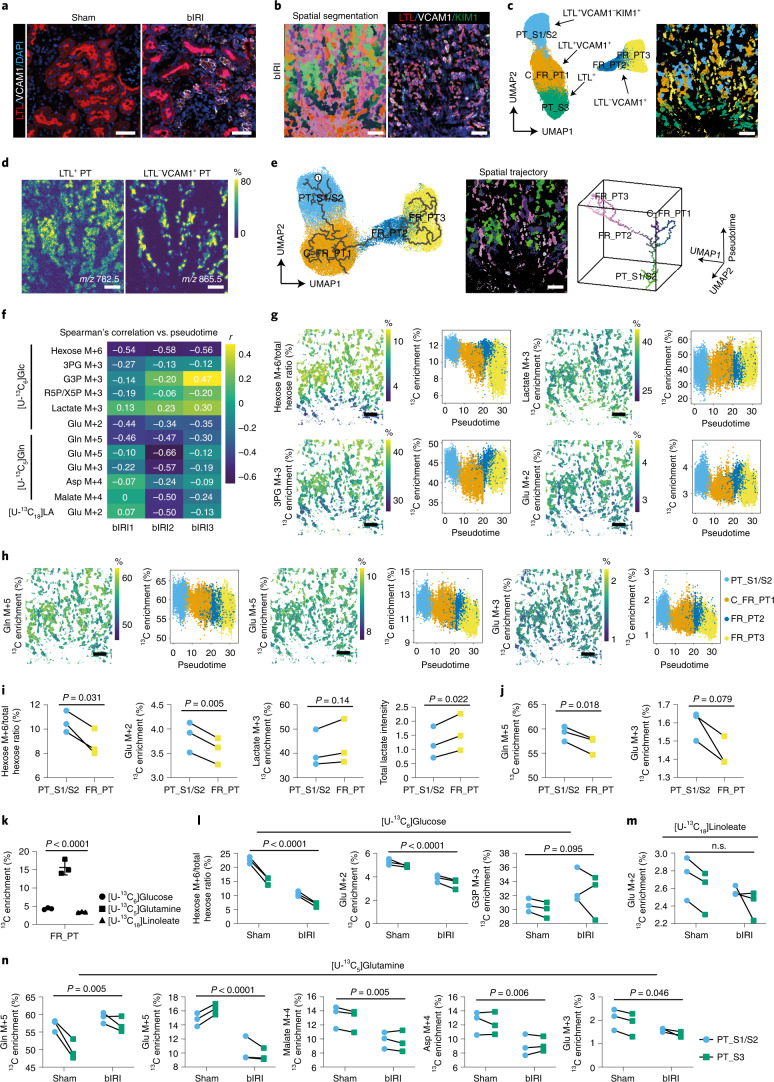
Dynamic metabolic measurements on bIRI kidney PT cells. **a**, Representative images of LTL and VCAM1 IF staining on sham and bIRI kidneys (*n* = 3). **b**, Left, representative molecular histology of cortical and outer stripe outer medulla (left) areas of bIRI kidney (*n* = 3), generated from UMAP of recorded lipid profiles. Right, IF staining following MALDI-MSI. **c**, UMAP plot (left) and representative spatial segmentation (right) showing lipid heterogeneity between PT cells from cortical and OSOM of bIRI kidneys (*n* = 3). Spatial segmentation image showing the distribution of the UMAP clusters on tissue with same color. **d**, Representative lipid species distribution in cortical and outer stripe outer medulla areas of bIRI kidney (*n* = 3). **e**, Left, Embedding of PT pixels from bIRI kidneys (*n* = 3), showing the trajectory of PT injury using pseudotime lipidomics analysis (starting point 1). Middle, spatial-trajectory map showing the pseudotime score and UMAP embedding of each pixel on tissue. Right, 3D scatter plot of cell-type-specific trajectories, with the same colors as pixels in the spatial-trajectory map. **f**, Spearman’s correlation between pseudotime and average ^13^C isotopologue enrichment on all PT pixels. **g**,**h**, Dynamic metabolic measurements using [U-^13^C_6_]glucose (**g**) or [U-^13^C_5_]glutamine (**h**), and their trajectory changes on PT cells of bIRI kidneys (*n* = 3). **i**,**j**, Dynamic metabolic comparison of [U-^13^C_6_]glucose and total lactate levels (**i**) or [U-^13^C_5_]glutamine measurements (**j**) between LTL^+^VCAM1^–^KIM1^–^ PT cells (PT-S1/S2) and LTL^–^VCAM1^+^ PT cells (FR_PT). A two-tailed paired *t*-test was performed. **k**, Direct carbon contribution of different nutrients to glutamate at 2 hours in FR_PT, from the glutamate isotopologues M+2, M+3, and M+5. One-way ANOVA was performed (*n* = 3). Comparison of dynamic metabolic measurements using [U-^13^C_6_]glucose (**l**), [U-^13^C_18_]linoleate (**m**), or [U-^13^C_5_]glutamine (**n**) on PTs from sham kidneys and healthy PTs from IRI kidneys. Two-way ANOVA was performed. Images and graphs show the average ^13^C enrichment (area under curve (AUC) normalized to total time) of isotopologues derived from [U-^13^C_6_]glucose or [U-^13^C_5_]glutamine, measured at different timepoints (0, 15, 30, 60, and 120 minutes), or [U-^13^C_18_]linoleate, measured at different timepoints (0, 60, and 120 minutes). Scale bars: (**a**) 50 µm, (**b**–**e**,**g**,**h**) 200 µm. Source data

**Extended Data Fig. 6:**
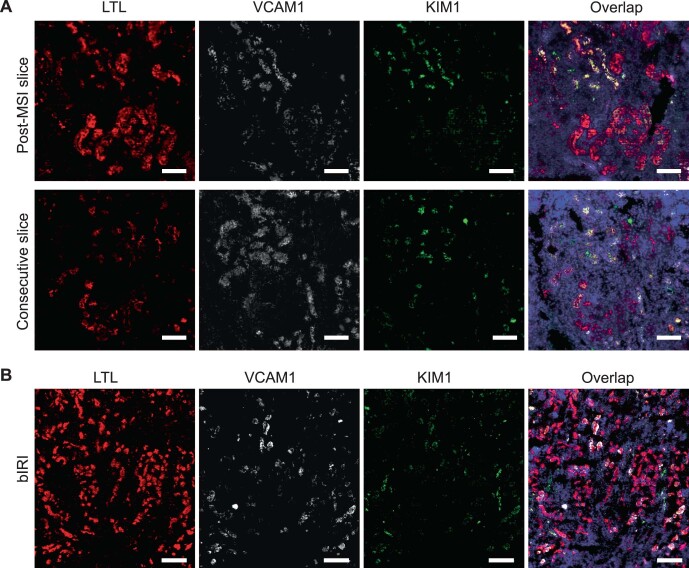
Immunofluorescence staining on mouse IRI kidneys. **a**, Validation of immunofluorescence staining of LTL, VCAM1 and KIM1 on the measured area of post-MSI slice compared to consecutive slice without MALDI matrix. Scale bar = 100 µm. **b**, Immunofluorescence staining of LTL, VCAM1 and KIM1 on bIRI kidney, same as the image shown in Fig. 4b. Scale bar = 200 µm.

We then sought to identify metabolic (mal)adaptation after bIRI by comparing healthy PT (LTL^+^VCAM1^–^) with failed maladaptive repaired PT (VCAM1^+^; FR_PT) (Fig. 4a). Following MALDI-MSI, we selected all pixels with a PT, determined on the basis LTL, VCAM1, and KIM1 IF staining (Fig. 4b and Extended Data Fig. 6). Four types of PT could subsequently be identified on the basis of lipid profiling: LTL^+^VCAM1^–^KIM1^–^ (PT-S1/S2) and LTL^+^VCAM1^ +^ (FR_PT1) in the cortex, and LTL^–^VCAM1^ +^ (FR_PT2 and FR_PT3) and LTL^+^ in the outer stripe of the outer medulla (PT-S3) (Fig. 4c,d). The clusters present in the cortex areas showed a trajectory towards maladaptive repair on the basis of their lipid profiles (Fig. 4e). The spatial-trajectory map reflected progressive maladaptive repair in different areas (Fig. 4e), consistent with loss of LTL staining and increased VCAM1 expression. Notably, FR_PT and normal PT were localized in the same region (Fig. 4e), indicating that maladaptive failed repair could not be explained by a differential impact of only the ischemic insult. We then calculated the correlation of pseudotime scores with ^13^C enrichment of several isotopologues (Fig. 4f). Following the trajectory from healthy PT areas toward progressively impaired PT areas, the pseudotime score was negatively correlated with [U-^13^C_6_]hexose enrichment (mean *r* = –0.56, *P* < 0.001) and its downstream product [^13^C_2_]glutamate (mean *r* = –0.38, *P* < 0.001) but was weakly positively correlated with [^13^C_3_]lactate enrichment (mean *r* = 0.22, *P* < 0.001), in combination with more total lactate present in FR_PT (Fig. 4j). One possible explanation for this is a higher glycolytic flux to the production of lactate and a lower contribution of [U-^13^C_6_]glucose to the TCA cycle in FR_PT (Fig. 4f,g,i), although it cannot be ruled out that other sources contribute to total lactate. A reduction of both [U-^13^C_5_]glutamine enrichment and labeled-glutamine-derived isotopologues through the oxidative TCA cycle was also observed (Fig. 4f,h,j), but glutamine, compared with glucose and linoleate, remained the main carbon source for glutamate in FR_PT (Fig. 4k). From these data, we conclude that the maladaptive repair in the PT is characterized by differences in production of lactate, which could be possibly the result of higher glycolytic activity and, as injury progresses, a concomitant reduction of TCA cycle metabolite consumption.

We then focused on normal PTs in the bIRI kidneys. Strikingly, when compared with the sham kidneys, these epithelial cells still displayed abnormal nutrient partitioning to TCA metabolism, both in the cortex as well as in the outer medulla (Fig. 4l,m). Normal PTs, 2 weeks after bIRI, had lower [U-^13^C_6_]hexose and [^13^C_2_]glutamate enrichment than sham kidneys (Fig. 4l). In addition, although normal PTs exhibited higher [U-^13^C_5_]glutamine enrichment, we found a lower ^13^C enrichment in specific isotopologues that are produced through the oxidative TCA cycle, such as [U-^13^C_5_]glutamate, [^13^C_4_]malate, [^13^C_4_]aspartate, and [^13^C_3_]glutamate (Fig. 4n). This is remarkable, as the TCA is a central hub in cell function and a cell’s ability to adapt to its environment. Its metabolites not only serve in the biosynthesis of nucleotides and macromolecules such as lipids and proteins, but also have recently been recognized to control gene regulation through post-translational histone modifications and DNA methylation^19^; hence, it will be of relevance to study how the disbalance of TCA metabolite homeostasis, even in normal PT cells after bIRI, relates to chronic progressive kidney injury or loss of repair after a new injury.

**Extended Data Fig. 7:**
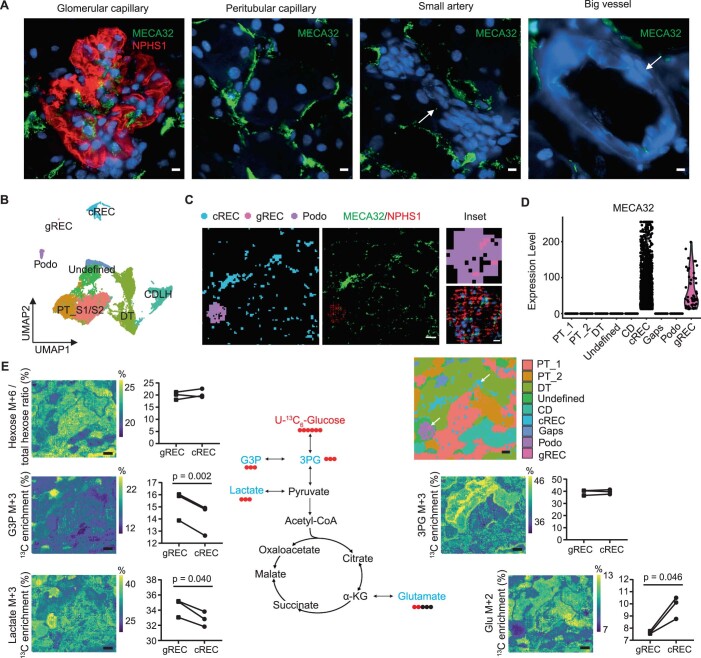
Dynamic metabolic measurements on kidney endothelial cells. **a**, MECA32 staining of different vessels in mouse kidney to show specific capillary endothelial cell staining (Scale bar = 5 µm). Arrows depict the vessels with negative MECA32 staining. **b**, Metabolic heterogeneity within cortical cells in kidney, visualized in a two-dimensional UMAP plot of combined MALDI-MSI data and immunofluorescence staining at 5 × 5 µm^2^ pixel-size. Glomerular endothelial cells (gREC) and peritubular endothelial cells (cREC) were identified using the post-MALDI-MSI anti-MECA32 antibody staining and podocytes (Podo) with anti-NPHS1 antibody staining. Proximal tubules (PT), distal tubules (DT) and collecting ducts (CD) were identified based on their respective lipid profiles obtained from measurement shown in Extended Data Fig. 1b. **c**, Immunofluorescent (MECA32 and NPHS1, middle panel; scale bar = 40 µm) staining on post-MALDI-MSI tissue to determine gREC, cREC and podocyte distribution (left panel). Pixel colors used are similar to the given UMAP cell-cluster colors in A. Inset view of detailed glomerular area (right panels, scale bar = 5 µm). **d**, MECA32 expression in the observed cell types. **e**, Dynamic metabolic measurements using U-^13^C_6_-glucose. Graphs show the ^13^C enrichment of isotopologues of glucose, glycerol 3-phosphate (G3P), 3-phosphoglycerate (3PG), lactate and glutamate over time in gREC and cREC. Two tailed paired t-test was performed (n = 3). Spatial UMAP shows the metabolic cellular architecture of the tissue (arrows depict cREC and gREC). Pixel colors used are similar to the given UMAP cell-cluster colors in B. Scale bar = 200 µm. Source data

The dynamic nature of metabolism, and the metabolic heterogeneity of different cell types and phenotypes within the architecture of a single tissue, call for single-cell-resolution measurements of metabolic dynamics. Currently available methods can provide singular spatial metabolic snapshots, bulk dynamic metabolism of homogenized tissue material, or isotope tracing on tissue with low spatial resolution^1,2,6–9^. Other strategies, based on single-cell transcriptomics^20^, cytometry by time of flight (CyTOF)^21^, or metabolic probes^22^, have yielded information on single-cell metabolism. However, these methods are based on the detection of surrogates for metabolic activity, such as metabolic enzymes and their transcripts. Here, we provide an in situ cell-type-specific measurement of metabolic dynamics through the direct detection of metabolic intermediates at single-cell resolution. Furthermore, to detect the dynamic metabolism of less abundant cell types in tissue, such as endothelial or immune cells, we introduced IF staining values to correlate with lipid profiles as a threshold for cell selection. For example, the combination of IF staining and lipid profiles allowed us to identify two populations of renal cortex endothelial cells: glomerular renal endothelial cell (gRECs) and cortical renal endothelial cells (cRECs) (Extended Data Fig. 7a–d). In line with known continuous VEGF signaling from podocytes^23^, gRECs showed higher [^13^C_3_]lactate enrichment than cRECs (Extended Data Fig. 7e). The combination of multiplexed IF staining on post-MSI-analyzed tissues with MALDI-MSI data shows great potential for multi-omics analysis by combining dynamic metabolomics with antibody-based spatial proteomics^24,25^. This will link dynamic metabolism to the cell genotype and phenotype, giving more insight into metabolic regulated cell behavior. The accuracy of image co-registration of the spatial distribution of clustered pixels with IF staining, as in the bIRI study in this paper, is less critical than co-registration at the single-pixel level. To combine IF staining values with an MSI lipid dataset, a more advanced method for co-registration could be used^26^. A single-cell segmentation step based on IF can also be used to study dispersed single-cell populations in tissues, such as immune cells^27^. Although in this study we used pixels for UMAP and trajectory analysis, rather than individual cells after single-cell segmentation, both cell typing and trajectory were in line with IF staining, which indicates that single-cell-resolution pixels can be used for cell-type-specific analysis, as has been previously shown using spatial transcriptomics technologies^28,29^.

**Extended Data Fig. 8:**
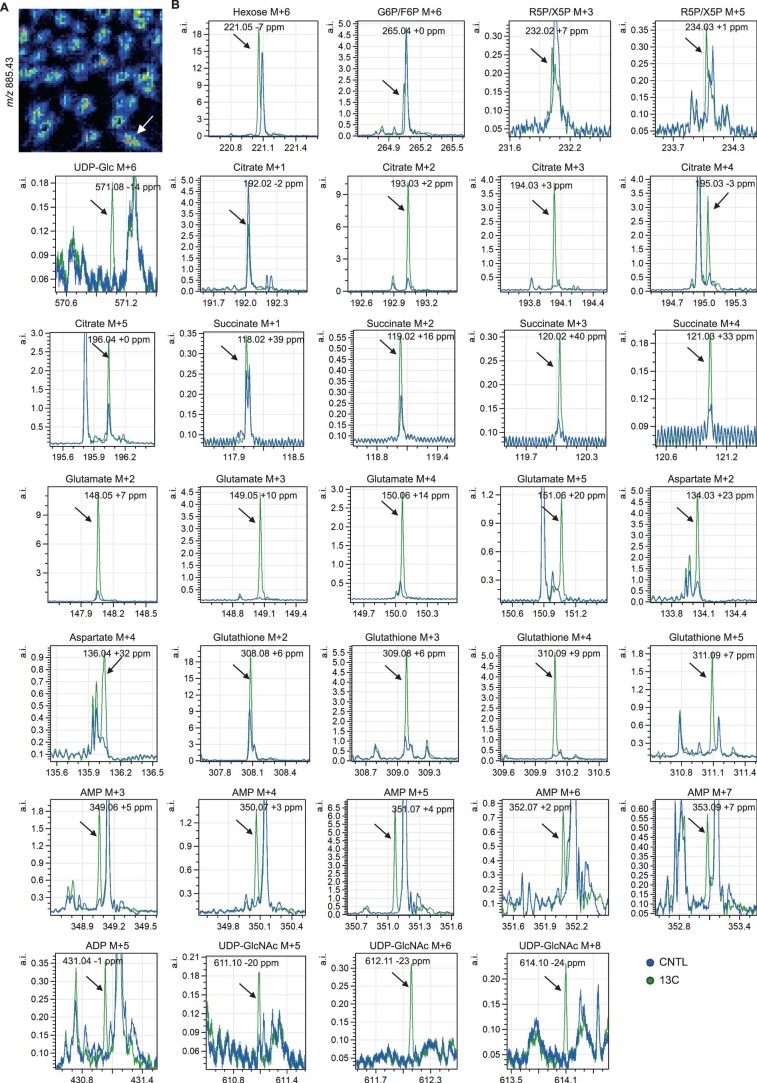
High spatial resolution MALDI-MSI measurement on cultured endothelial cell. **a**, MALDI-MSI data from measurements of cultured endothelial cells, which were incubated with U-^13^C_6_-glucose for 24 h. Arrow depicts a single endothelial cell. **b**, Mass spectra obtained from ^13^C-labeled- and control samples measured by MALDI-MSI at 5 × 5 µm^2^ pixel size to show the presence of ^13^C-labeled metabolites.

To make this method potentially usable in human tissue biopsies, we used vibratome sectioning for stable-isotope tracing on tissue ex vivo, which provides the possibility of tracing different isotopes using a single tissue sample. However, the current 2-hour incubation period limits the dynamic measurement to central carbon metabolic pathways (glycolysis and TCA cycle) in metabolic non-steady state, as well as the detection of ^13^C-labeled TCA intermediates from one oxidative cycle. Nonetheless, this MALDI-MSI-based method for cell-type-specific measurement of metabolic dynamics can be applied to tissue samples or cultured cells for which longer incubation of stable isotopes is feasible, thus enabling the detection of ^13^C-labeled metabolites in a pseudo-steady-state. For instance, in endothelial cells cultured in vitro for 24 hours in the presence of [U-^13^C_6_]glucose, we could detect not only the full range of isotopologues of TCA intermediates, such as citrate (M+1, M+2, M+3, M+4, and M+5) and succinate (M+1, M+2, M+3, and M+4), but also^13^C-labeled metabolites from other metabolic pathways, such as ribose-5-phosphate/xylulose-5-phosphate (R5P/X5P), adenosine monophosphate (AMP), adenosine diphosphate (ADP), uridine diphosphate glucose (UDP-Glc), uridine diphosphate *N*-acetylglucosamine (UDP-GlcNAc), and glutathione (Extended Data Fig. 8). Owing to the limitations of MALDI-MS, we could not distinguish isomeric molecular species, such as glucose, galactose, mannose, and inositol^9^, as well as the confounders interfering with the quantification of labeling peaks, such as [^13^C_2_]aspartate and [^13^C_2_]malate. To solve this, more advanced instrumentation using ion-mobility separation is needed^30^. Delocalization of metabolites is a limitation of spatial metabolomics with MALDI-MSI in general, which could cause false negatives by decreasing the signal differences between cell types, but is less likely to cause false positives^31^.

In summary, we have developed a platform to detect cell-type-specific dynamic metabolic changes at single-cell resolution. We demonstrated that this platform, applied to tissue samples, can discern metabolic heterogeneity within individual cell types in relation to the microenvironment. We used the kidney as an example of a complex and metabolically heterogenous organ, but this method can be used to investigate tissue homeostasis in general. Our method could be applied to detect changes in cell-specific metabolism, which occur in processes such as inflammation, fibrosis, and cancer biology.

## Methods

### Reagents

All reagents were obtained from Sigma-Aldrich, unless stated otherwise.

### Mouse studies

For studies of overall metabolic changes, post mortem material of 12-week-old male C57BL/6J mice (*n* = 3) culled as breeding surplus were used. Mice were kept and cared for in accordance with the Experiments on Animals Act (Wod, revision 2014, the Netherlands) and EU directive no. 2010/63/EU. Mice were housed at 20–22 °C in individually ventilated cages, humidity controlled (55%) with free access to food and water and a light/dark cycle of daytime (06:30–18:00) and nighttime (18.00–06:30).

For bIRI) experiments, we used 12-week-old male constitutional renin reporter (B6.Ren1cCre/TdTomato/J) mice^32^. Six mice were divided into two groups randomly (*n* = 3/group). In short, mice were placed under isoflurane anesthesia and controlled body temperature was kept at 36.7 °C, and renal arteries and veins were exposed through laparotomy with median incision on both sides. Subsequently, both arteries and veins were ligated using clamps for approximately 18 minutes, after which the clamps were removed to resume blood flow (reperfusion) and the abdomen was closed. For the sham controls, mice were opened up and closed similarly, but no ligations were performed. At day 14 after surgery, mice were perfused with cold PBS–heparin (5 IU/mL) via the left ventricle at a controlled pressure of 150 mmHg for 6 minutes to exsanguinate the kidneys before removal. Animal experiments were approved by the Ethical Committee on Animal Care and Experimentation of the Leiden University Medical Center (permit no. AVD1160020171145).

### Vibratome sectioning and tissue slice incubation

Directly after euthanization, kidneys were collected and kept in ice-cold sterile Hanks’ Balanced Salt Solution (HBSS) with 5 mM glucose and penicillin–streptomycin before vibratome sectioning. Each kidney was embedded in 4% low-melting-temperature agarose gel, and 350-µm-thick tissue slices were obtained from fresh tissue under ice-cold HBSS with 5 mM glucose and penicillin–streptomycin using a Vibratome VT1200 (Leica Microsystems). Slicing speed was 0.1 mm/second, and vibration amplitude was 2 mm.

Tissue slices were placed into culture plates and incubated in a well-defined medium (nutrients-free Seahorse XF DMEM assay medium (Agilent, 103681), supplemented with 2% FCS, 3 mM linoleic acid (dissolved with addition of 1% BSA), 5 mM glucose, 500 µM glutamine, 100 µM sodium acetate, 50 µM sodium citrate, and penicillin–streptomycin (pH adjusted to 7.4)) for up to 2 hours at 37 °C and 5% CO_2_. During incubation, medium was changed to media containing various ^13^C-labeled nutrients at different time points. For the ^13^C-labeling incubation, the same amounts of either [U-^13^C_6_]glucose (99%, Sigma, 389374), [U-^13^C_6_]glutamine (99%, Cambridge Isotope Laboratories, Inc. CLM-1822-H), or [U-^13^C_18_]linoleic acid (98%, Cambridge Isotope Laboratories, CLM-6855-0) were used to replace similar unlabeled nutrients in each medium. In the end, tissue slices were quenched with liquid N_2_ and stored at −80 °C.

### Tissue preparation and matrix deposition

Tissue slices were embedded in 10% gelatin and cryosectioned into 10-µm-thick sections using a Cryostar NX70 cryostat (Thermo Fisher Scientific) at –20 °C. The sections were thaw-mounted onto indium-tin-oxide (ITO)-coated glass slides (VisionTek Systems). Mounted sections were placed in a vacuum freeze-dryer for 15 minutes prior to matrix application. After drying, *N*-(1-naphthyl) ethylenediamine dihydrochloride (NEDC) (Sigma-Aldrich, UK) MALDI-matrix solution of 7 mg/mL in methanol/acetonitrile/deionized water (70, 25, 5% vol/vol/vol) was applied using a SunCollect sprayer (SunChrom). A total of 21 matrix layers were applied with the following flow rates: layer 1–3 at 5 µL/min, layer 4–6 at 10 µL/min, layer 7–9 at 15 µL/min and 10–21 at 20 µL/min (speed *x*, medium 1; speed *y*, medium 1; *z* position, 35).

### MALDI-MSI measurement

MALDI-TOF/TOF-MSI was performed using a RapifleX MALDI-TOF/TOF system (Bruker Daltonics). Negative-ion-mode mass spectra were acquired at a pixel size of 5 × 5 µm^2^ or 20 × 20 µm^2^ over a mass range of *m/z* 80–1000. Prior to analysis, the instrument was externally calibrated using red phosphorus. Spectra were acquired with 15 laser shots per pixel (for 5 µm measurement) or 200 laser shots per pixel (for 20 µm measurement) at a laser repetition rate of 10 kHz. Data acquisition was performed using flexControl (Version 4.0, Bruker Daltonics) and visualizations were obtained from flexImaging 5.0 (Bruker Daltonics). All the samples from same slides were measured randomly. MALDI-FTICR-MSI was performed on a 12 T solariX FTICR mass spectrometer (Bruker Daltonics) in negative-ion mode, using 30 laser shots and 50-µm pixel size. Prior to analysis, the instrument was calibrated using red phosphorus. The spectra were recorded over a *m/z* range of 100–1,000 with a 2M data point transient and transient length of 0.5592 seconds. Data acquisition was performed using ftmsControl (Version 2.1.0, Bruker Daltonics), and visualizations were obtained from flexImaging 5.0 (Bruker Daltonics). The images of lipid and metabolite distributions on tissue were exported from flexImaging 5.0. Following the MALDI-MSI data acquisition, excess matrix was removed by washing in 100% ethanol (2 × 5 min), 75% ethanol (1 × 5 min), and 50% ethanol (1 × 5 min), after which this MSI-analyzed tissue section was used for IF staining, as described below.

### Immunofluorescence staining

After MALDI-MSI tissues on the slide were fixed using 4% paraformaldehyde for 30 minutes, antigen retrieval was performed using antigen retrieval buffer (Dako, Agilent Technologies) in an autoclave. Slides were blocked with 3% normal donkey serum, 2% BSA, and 0.01% Triton X-100 in PBS for 1 hour at room temperature. Primary anti-mouse-KIM1 antibody (5 µg/mL, R&D Systems, MAB1817), anti-VCAM1 antibody (1:250, Abcam, ab134047), anti-CDH1 antibody (1:300, BD Biosciences, 610181), anti-NPHS1 (2 µg/mL, R&D Systems, AF4269), anti-mouse-pan-endothelial-cell-antigen (MECA32, 2 µg/mL, BD Biosciences, 553849), *Lotus tetragonolobus* Lectin (LTL, 1:300, Vector Laboratories, B-1325), or lectin from *Bandeiraea simplicifolia* isolectin B4 (BS1-TRITC, 1:200, Sigma, L5264) were incubated overnight at 4 °C, followed by corresponding fluorescent-labeled secondary antibodies (donkey anti-rat-IgG AF488 (1:300, Invitrogen, A21208), donkey anti-rabbit-IgG AF647 (1:300, Invitrogen, A31573), donkey anti-mouse-IgG AF488 (1:300, Invitrogen, A21202), donkey anti-sheep-IgG AF568 (1:300, Invitrogen, A21099), streptavidin–Alexa Flour 568 (1:300, Invitrogen, S11226), streptavidin–Alexa Flour 647 (1:300, Invitrogen, S32357)) for 1 hour at 4 °C, when necessary. Slides were embedded in Prolong gold antifade mountant with DAPI (Thermo Fisher Scientific, P36931). Fluorescent images of the slides were recorded using a 3D Histech Pannoramic MIDI Scanner (Sysmex). Digital scanned images were co-registered to the MALDI-MSI data.

### MSI data processing and analysis

**Extended Data Fig. 9:**
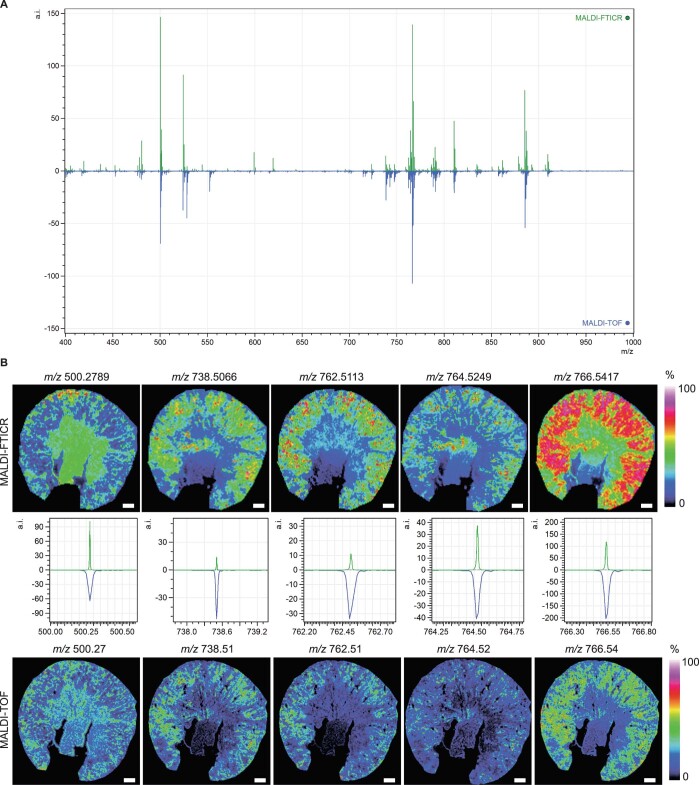
Validation of MALDI-FTICR and MALDI-TOF data. **a**, Comparison of the average mass spectrum of an area with 50 × 50 µm^2^ pixel-size (top, green) acquired by MALDI-FTICR and the average spectrum of 20 × 20 µm^2^ pixel-size (bottom, blue) acquired by MALDI-TOF. **b**, Comparison of the lipids distribution recorded by MALDI-FTICR at 50 × 50 µm^2^ pixel-size and MALDI-TOF at 20 × 20 µm^2^ pixel-size on same kidney. Scale bar = 500 µm.

MSI data were exported and processed in SCiLS Lab 2016b (SCiLS, Bruker Daltonics) with baseline correction using convolution algorithm. All MALDI-TOF-MSI data were normalized to the total ion count (TIC). Peak picking was performed (signal-to-noise ratio > 3) on the average spectrum, and matrix peaks were excluded from the *m/z* feature list. The *m/z* features which were present in both MALDI-FTICR-MSI and MALDI-TOF-MSI datasets, and which had similar tissue distributions, were further used for identity assignment of lipid species (Extended Data Fig. 9). The *m/z* values from MALDI-FTICR-MSI were imported into the Human Metabolome Database^33^ (https://hmdb.ca/) after re-calibration in mMass and annotated for lipids species with an error < ±5 ppm. For the small molecules detected only in MALDI-TOF, the *m/z* values from MALDI-TOF were imported into the Human Metabolome Database (https://hmdb.ca/) after re-calibration in mMass and annotated for metabolites with an error < ±20 ppm. The ^13^C-labeled peaks were selected by comparing the spectrum of control and ^13^C-labeling experiments, and annotated on the basis of the presence of unlabeled metabolites and their theoretical *m/z* values. For measurement at 20 × 20 µm^2^ pixel size, a total of 230 high-molecular-weight features (*m/z* > 400, predominantly phospholipids^12^, Extended Data Table 1) that not co-localized with matrix peaks were selected (signal-to-noise ratio > 3). For measurement at 5 × 5 µm^2^ pixel size, a total of 16 metabolites (9 unlabeled and 11 ^13^C-labeled), 227 high-molecular-weight features that were not co-localized with matrix peaks were selected (signal-to-noise ratio > 3). Peak intensities of the selected features were exported for all the measured pixels from SCiLS Lab 2016b, which were used for the following analysis. Natural isotope abundance correction was performed for metabolites using R package IsoCorrectoR^34^.

For targeted endothelial cell analysis at 5 × 5 µm^2^ pixel size, the high-resolution staining image of MECA32 and NPHS1 on the MALDI-MSI measured area was exported from CaseViewer (3DHISTECH). Staining in the areas not measured by MALDI-MSI was removed. The resolution of the exported image was changed to 5 × 5 µm^2^ pixel size using Matlab R2019a to comply with the MALDI-MSI resolution. By merging it with the previous high-resolution image, the pixels fully covering MECA32 staining were selected as endothelial cells, and non-fully-covered pixels were discarded from the image. Next, the staining values of all the pixels were exported using Matlab R2019a with a cutoff of 5% (values < 13 were replaced with 0). In the end, the staining values corresponding to the same pixels as MALDI-MSI measurements were integrated into MALDI-MSI data and used for data analysis as described below.

For UMAP analysis, the datasets were transformed into a count matrix by multiplying the TIC-normalized intensities by 100 and taking the integer. This count data matrix was normalized and scaled using SCTransform to generate a 2D UMAP map using Seurat 3.0 in R (version 4.0). The distribution of the pixels from different clusters on tissues were co-registered to the IF staining, and cell types were identified on the basis of both staining and their morphology on the kidney. Using measurements of endothelial cells, two endothelial cell clusters were identified on the basis of MECA32 staining and their position, and a podocyte cluster was identified on the basis of NPHS1 staining. For data imputation, the metabolite *m/z* features from the ‘control,’ or unlabeled, dataset were removed, so only lipid *m/z* features were left, which were used as the query. Then, the MALDI-MSI data from ^13^C-labeling experiments were used as a reference to transfer metabolite production into the query using FindTransferAnchors and TransferData function from the Seurat 3.0 package in R. Both the query and reference were normalized and scaled using SCTransform. Ultimately, all the imputed metabolite productions were combined into one dataset, which contained the ^13^C-labeling information over time.

After combining all the imputed data into one dataset, the fraction enrichment of isotopologues was calculated on the basis of the ratio of each ^13^C-labeled metabolite (isotopologue) to the sum of this metabolite abundance in each pixel. The calculated fraction enrichment of isotopologues was used to generate pseudoimages together with pixel coordinate information exported from SCiLS Lab 2016b. The average fraction enrichment values (AUC normalized to total time) of identified clusters were used for generating graphs and statistical analysis. Hotspot removal (high quantile 99%) were applied to all the pseudoimages generated from calculated values.

The integrated data matrix from 3 IRI kidneys from Seurat 3.0 was further used to for the trajectory analysis using Monocle3 (ref. ^35^) in R. A spatial trajectory map was generated using Matlab R2019a based on embedding information of UMAP and pseudotime values calculated by Monocle3 and pixel coordinate information exported from SCiLS Lab 2016b as descripted below.

For spatial UMAPs, the datasets were used to generate a 3D UMAP map using packages Seurat 3.0 and plotly. The embedding information of the 3D UMAP was translated to RGB color coding by varying red, green and blue intensities on the three independent axes. Together with pixel coordinate information exported from SCiLS Lab 2016b, a MxNx3 data matrix was generated and used to generate UMAP images in Matlab R2019a.

Data collection and analysis were not performed blind to the conditions of the experiments. A step-by-step protocol associated with this study is available in Protocol Exchange (10.21203/rs.3.pex-1912/v1)^36^.

### Validation test of imputation performance

Validation of the imputation performance using ‘leave-one factor-out’ cross-validation is shown in Extended Data Fig. 3. In short, the metabolite production from MALDI-MSI data was taken out from one kidney slice (sample 1, Extended Data Fig. 3a) or two different kidney slices (sample 3, Extended Data Fig. 3h) and use samples 2 and 4 to impute the metabolite production in sample 1 and in sample 3, respectively, as described above. Next, Spearman’s correlation was calculated between the imputed and detected values in sample 1 and 3. Values higher than 0.8 were graded as a very strong positive correlation. Values between 0.6 to 0.8 were a strong positive correlation, and between 0.4 to 0.6 a moderate positive correlation.

### Statistics and reproducibility

No statistical methods were used to predetermine sample sizes, but our sample sizes are similar to those reported in previous publications^9,16^. All the experiments and data analysis were performed in triplicate (3 animals per group). No animals or data points were excluded from the analysis. All the data are presented as mean ± s.d., unless indicated otherwise. The average fractional contribution values of identified clusters were used for statistical analysis. AUC was used for comparison of the changes following timecourse of isotope tracer incubation up to 2 hours between different groups. Data normality and equal variances were tested using the Shapiro–Wilk test. Differences between groups were assessed by paired two-tailed Student’s *t*-test, two-tailed Student’s *t*-test, when not normally distributed, or by two-tailed *F*-test. *P* < 0.05 was considered statistically significant.

### Reporting summary

Further information on research design is available in the Nature Research Reporting Summary linked to this article.

### Supplementary information

Reporting Summary

### Source data

Source Data Fig. 2 Statistical Source Data

Source Data Fig. 3 Statistical Source Data

Source Data Fig. 4 Statistical Source Data

Source Data Extended Data Fig. 3 Statistical Source Data

Source Data Extended Data Fig. 4 Statistical Source Data

Source Data Extended Data Fig. 5 Statistical Source Data

Source Data Extended Data Fig. 7 Statistical Source Data

## Figures and Tables

**Extended Data Table 1:**
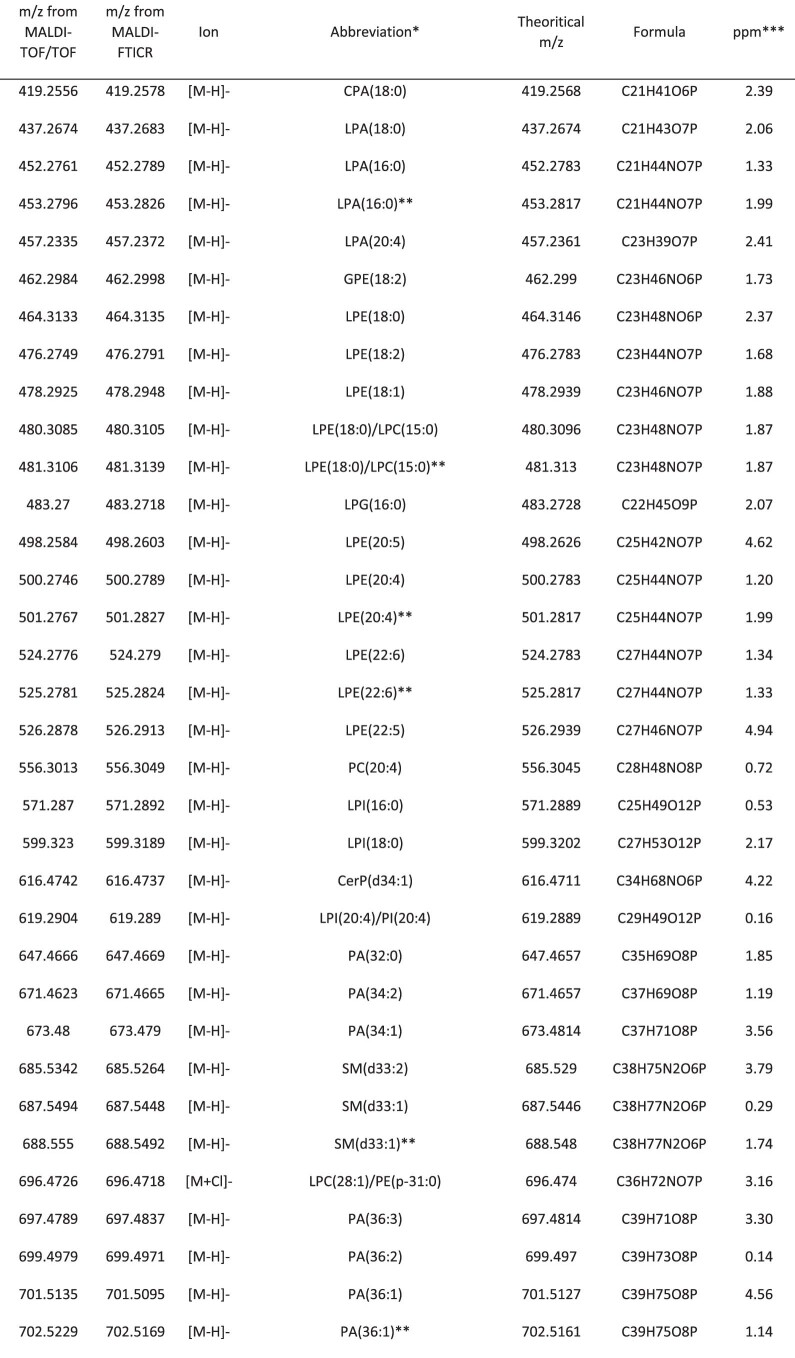

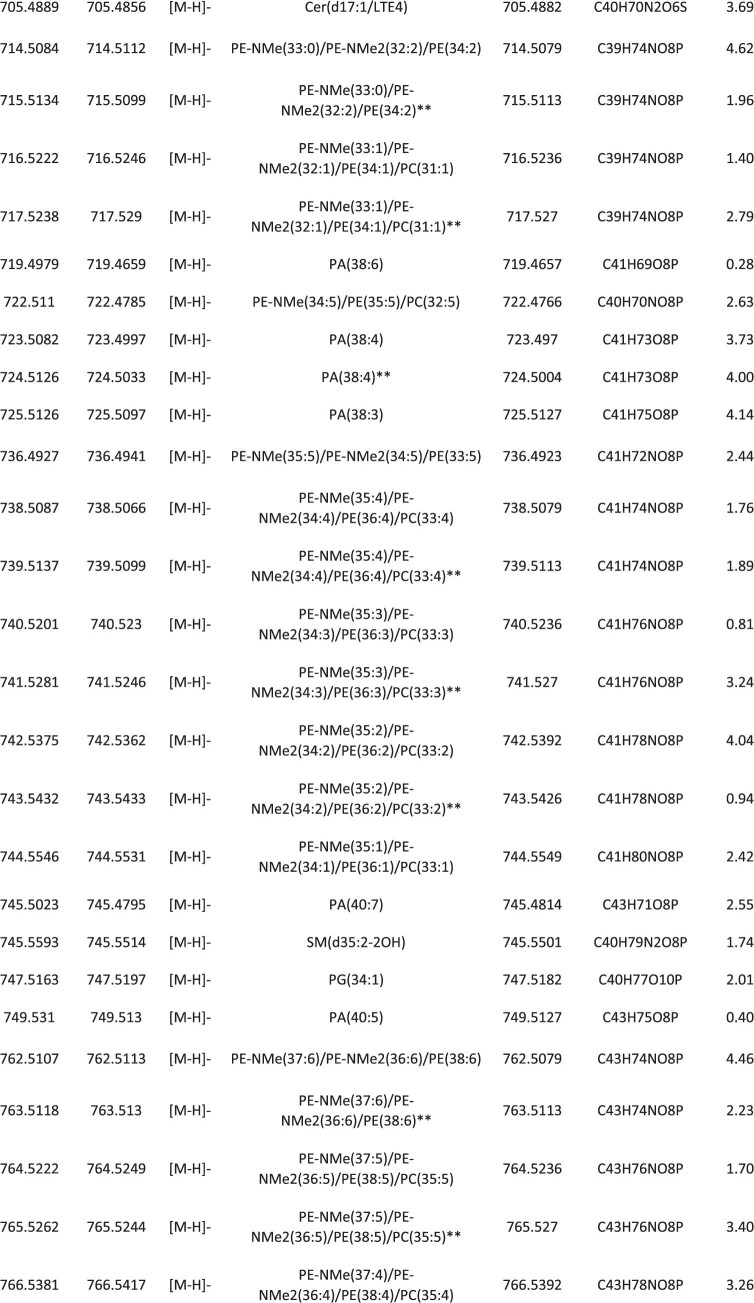

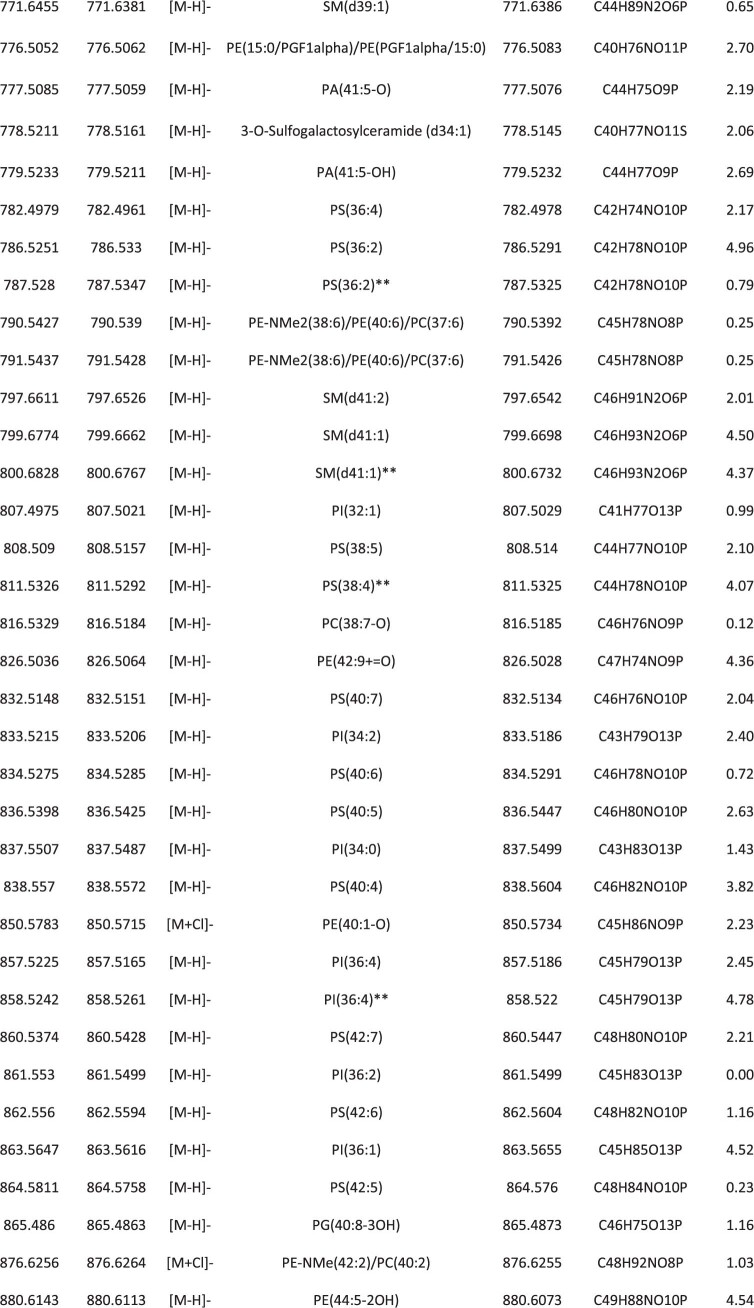

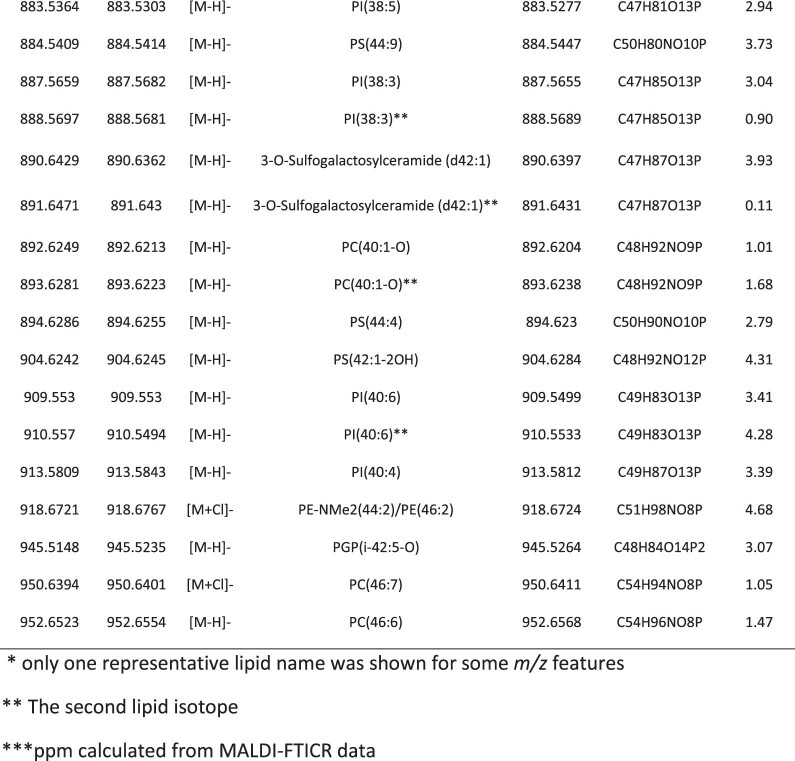
Example of lipids in mouse kidney assigned by the mass measurements using both MALDI-TOF/TOF and MALDI-FTICR MS in negative ion mode

## Data Availability

The exported and processed MSI data for this study were deposited in FigShare at 10.6084/m9.figshare.20227419.v1. Owing to the large size of all raw data, parts of the raw MSI data are deposited to provide the necessary information of ^13^C-labeled metabolites and spectrum quality. For full raw MALDI-MSI data related to this study, please contact G. W. (g.wang@lumc.nl) or B. H. (b.p.a.m.heijs@lumc.nl). Upon reasonable request, data will be made available. Source data are provided with this paper.

## Code availability

The codes used in this article is available in Github (https://github.com/Gangqiwang/scDYMO).
