# Supplementary material for: Analyzing cell-type-specific dynamics of metabolism in kidney repair
Source: Nat Metab. 2022 Aug 25;4(9):1109–18. doi: 10.1038/s42255-022-00615-8 (PMC9499864; doi:10.1038/s42255-022-00615-8)
Supplement: Supplementary file 1 — Reporting Summary [file 42255_2022_615_MOESM1_ESM.pdf]

nature portfolio

Corresponding author(s): Gangqi Wang
Last updated by author(s): Jul 5, 2022

Reporting Summary

Nature Portfolio wishes to improve the reproducibility of the work that we publish. This form provides structure for consistency and transparency in reporting. For further information on Nature Portfolio policies, see our Editorial Policies and the Editorial Policy Checklist.

Statistics

For all statistical analyses, confirm that the following items are present in the figure legend, table legend, main text, or Methods section.

n/a	Confirmed	
☐	☒	The exact sample size (n) for each experimental group/condition, given as a discrete number and unit of measurement
☐	☒	A statement on whether measurements were taken from distinct samples or whether the same sample was measured repeatedly
☐	☒	The statistical test(s) used AND whether they are one- or two-sided<br>*Only common tests should be described solely by name; describe more complex techniques in the Methods section.*
☐	☒	A description of all covariates tested
☐	☒	A description of any assumptions or corrections, such as tests of normality and adjustment for multiple comparisons
☐	☒	A full description of the statistical parameters including central tendency (e.g. means) or other basic estimates (e.g. regression coefficient) AND variation (e.g. standard deviation) or associated estimates of uncertainty (e.g. confidence intervals)
☐	☒	For null hypothesis testing, the test statistic (e.g. F, t, r) with confidence intervals, effect sizes, degrees of freedom and P value noted<br>*Give P values as exact values whenever suitable.*
☒	☐	For Bayesian analysis, information on the choice of priors and Markov chain Monte Carlo settings
☐	☒	For hierarchical and complex designs, identification of the appropriate level for tests and full reporting of outcomes
☐	☒	Estimates of effect sizes (e.g. Cohen's d, Pearson's r), indicating how they were calculated

Our web collection on statistics for biologists contains articles on many of the points above.

Software and code

Policy information about availability of computer code

Data collection	flexControl (Version 4.0, Bruker Daltonics); ftmsControl (Version 2.1.0, Bruker Daltonics);
Data analysis	flexImaging 5.0 (Bruker Daltonics); SCiLS Lab 2016b (SCiLS, Bruker Daltonics);CaseViewer (version 2.4, 3DHISTECH Ltd); Matlab R2019a; R (version 4.0); Seurat 3.0; Matlab R2019a; Monocle 3; RStudio (version 1.4.1717); pheatmap (version 1.0.12); IsoCorrectoR (version 1.14.0)<br>The code used in this study is available in Github (https://github.com/Gangqiwang/scDYMO).

For manuscripts utilizing custom algorithms or software that are central to the research but not yet described in published literature, software must be made available to editors and reviewers. We strongly encourage code deposition in a community repository (e.g. GitHub). See the Nature Portfolio guidelines for submitting code & software for further information.

Data

Policy information about availability of data

All manuscripts must include a data availability statement. This statement should provide the following information, where applicable:
- Accession codes, unique identifiers, or web links for publicly available datasets
- A description of any restrictions on data availability
- For clinical datasets or third party data, please ensure that the statement adheres to our policy

The exported and processed MSI data for this study were deposited in FigShare at https://doi.org/10.6084/m9.figshare.20227419.v1. Due to the large size of all raw data, parts of the raw MSI data are deposited to provide the necessary information of 13C-labeled metabolites and spectrum quality. For full availability of raw MALDI-MSI data related to this study, please contact Gangqi Wang (g.wang@lumc.nl) or Bram Heijs (b.p.a.m.heijs@lumc.nl) upon reasonable request data will be made available. Source data are provided with this article.
Human Metabolome Database (https://hmdb.ca/) was used for lipid and metabolite annotation.

Field-specific reporting

Please select the one below that is the best fit for your research. If you are not sure, read the appropriate sections before making your selection.

- [x] Life sciences
- [ ] Behavioural & social sciences
- [ ] Ecological, evolutionary & environmental sciences

For a reference copy of the document with all sections, see nature.com/documents/nr-reporting-summary-flat.pdf

Life sciences study design

All studies must disclose on these points even when the disclosure is negative.

Sample size	For overall metabolic changes post mortem material of 12-week-old male C57BL/6J mice (n = 3) culled as breeding surplus was used. For bilateral ischemia and reperfusion injury experiments (bIRI) we used 12-week-old male constitutional renin reporter (B6.Ren1cCre/TdTomato/J) mice. 6 mice were divided into 2 groups randomly (n = 3/group). No statistical methods were used to pre-determine sample sizes, but our sample sizes are similar to those reported in previous publications.
Data exclusions	No data was excluded from the analyses.
Replication	All the experiments and data analysis were performed on 3 biological replicates (3 animals per group).
Randomization	For bIRI experiment, 6 mice were divided into 2 groups randomly (n = 3/group).
Blinding	This is not relevant in this study, since there was no group allocation.

Reporting for specific materials, systems and methods

We require information from authors about some types of materials, experimental systems and methods used in many studies. Here, indicate whether each material, system or method listed is relevant to your study. If you are not sure if a list item applies to your research, read the appropriate section before selecting a response.

Materials & experimental systems

n/a	Involved in the study	
[ ]	[x]	Antibodies
[x]	[ ]	Eukaryotic cell lines
[x]	[ ]	Palaeontology and archaeology
[ ]	[x]	Animals and other organisms
[x]	[ ]	Human research participants
[x]	[ ]	Clinical data
[x]	[ ]	Dual use research of concern

Methods

n/a	Involved in the study	
[x]	[ ]	ChIP-seq
[x]	[ ]	Flow cytometry
[x]	[ ]	MRI-based neuroimaging

Antibodies

Antibodies used	Monoclonal Rat IgG2B anti-mouse KIM1 antibody (5 µg/ml, R&D Systems, Abingdon, UK, MAB1817), Rabbit monoclonal anti-VCAM1 antibody (1:250, abcam, ab134047); Mouse IgG2a anti-CDH1 antibody (1:300, BD Biosciences, 610181); Polyclonal Goat IgG anti-NPHS1 (2 µg/mL, R&D Systems, AF3159); Rat IgG2a anti-mouse pan-endothelial cell antigen (2 µg/mL, MECA32, BD Biosciences, 553849); donkey anti rat IgG AF488 (1:300, Invitrogen, A21208); donkey anti rabbit IgG AF647 (1:300, Invitrogen, A31573); donkey anti mouse IgG AF488 (1:300, Invitrogen, A21202); donkey anti sheep IgG AF568 (1:300, Invitrogen, A21099);
Validation	anti-mouse KIM1 antibody (R&D Systems, Abingdon, UK, MAB1817): source: Monoclonal Rat IgG2B Clone # 222414; Species Reactivity: mouse; Specificity: Detects mouse TIM 1/KIM-1/HAVCR in ELISAs and Western blots. This antibody does not cross-react with recombinant human (rh) TIM 1, rmTIM-2, or rmTIM-3; validation statements, relevant citation and other information can be found from the manufacturer's website: https://www.rndsystems.com/products/mouse-tim-1-kim-1-havcr-antibody-222414_mab1817<br>anti-VCAM1 antibody (abcam, ab134047): source: Rabbit monoclonal [EPR5047]; Species Reactivity: Mouse, Rat, Human; Application: WB, IP, IHC-P, Flow Cyt (Intra), ICC/IF, ELISA; validation statements, relevant citation and other information can be found from the manufacturer's website: https://www.abcam.com/VCAM1-antibody-EPR5047-ab134047.html?gclsrc=aw.ds|aw.ds&gclid=CjwKCAjwyryUBhBSEiwAGN5OCK-ODP6CXh8uHi9qIeNoHz46ftMFojX_C3Y3VRXYgTwehAZ76kSY8RoC3yoQAvD_BwE<br>anti-CDH1 antibody (BD Biosciences, 610181): source: Mouse IgG2a, κ; species reactivity: Human (QC Testing), Mouse, Rat, Dog (Tested in Development); Application: Western blot (Routinely Tested), Immunofluorescence, Immunohistochemistry, Immunoprecipitation (Tested During Development); validation statements, relevant citation and other information can be found from the manufacturer's website: https://www.bdbiosciences.com/en-us/products/reagents/microscopy-imaging-reagents/immunofluorescence-reagents/purified-mouse-anti-e-cadherin.610181.<br>anti-NPHS1 (R&D Systems, Abingdon, UK, AF3159): source: Polyclonal Goat IgG; species reactivity: Mouse; Application: Immunohistochemistry; validation statements, relevant citation and other information can be found from the manufacturer's

website: https://www.rndsystems.com/products/mouse-nephrin-antibody_af3159.
anti-mouse pan-endothelial cell antigen (MECA32, BD Biosciences, 553849): source: Rat IgG2a, κ; species reactivity: Mouse; Application: Flow cytometry (Routinely Tested), Immunohistochemistry-frozen, Immunoprecipitation, Western blot (Reported); validation statements, relevant citation and other information can be found from the manufacturer's website: https://www.bdbiosciences.com/en-us/products/reagents/western-blotting-and-molecular-reagents/western-blot-reagents/purified-rat-anti-mouse-panendothelial-cell-antigen.553849.
donkey anti rat IgG AF488 (Invitrogen, A21208): https://www.thermofisher.com/antibody/product/Donkey-anti-Rat-IgG-H-L-Highly-Cross-Adsorbed-Secondary-Antibody-Polyclonal/A-21208
donkey anti rabbit IgG AF647 (Invitrogen, A31573): https://www.thermofisher.com/antibody/product/Donkey-anti-Rabbit-IgG-H-L-Highly-Cross-Adsorbed-Secondary-Antibody-Polyclonal/A-31573
donkey anti mouse IgG AF488 (Invitrogen, A21202): https://www.thermofisher.com/antibody/product/Donkey-anti-Mouse-IgG-H-L-Highly-Cross-Adsorbed-Secondary-Antibody-Polyclonal/A-21202
donkey anti sheep IgG AF568 (Invitrogen, A21099): https://www.thermofisher.com/antibody/product/Donkey-anti-Sheep-IgG-H-L-Cross-Adsorbed-Secondary-Antibody-Polyclonal/A-21099

Animals and other organisms

Policy information about studies involving animals; ARRIVE guidelines recommended for reporting animal research

Laboratory animals	12-week-old male C57BL/6J mice and renin reporter (B6.Ren1cCre/TdTomato/J) mice.
Wild animals	the study did not involve wild animals
Field-collected samples	the study did not involve samples collected from the field
Ethics oversight	We used post mortem material of 12-week-old male C57BL/6J mice culled as breeding surplus. Mice were kept and cared for in accordance with the Experiments on Animals Act (Wod, revision 2014, The Netherlands) and EU directive no.2010/63/EU. For bIRI study, animal experiments were approved by the Ethical Committee on Animal Care and Experimentation of the Leiden University Medical Center (permit no. AVD1160020171145).

Note that full information on the approval of the study protocol must also be provided in the manuscript.
